# *SLFN14* gene mutations associated with bleeding

**DOI:** 10.1080/09537104.2019.1648781

**Published:** 2019-08-04

**Authors:** Rachel J. Stapley, Vera P. Pisareva, Andrey V. Pisarev, Neil V. Morgan

**Affiliations:** 1 Institute of Cardiovascular Sciences, College of Medical and Dental Sciences, University of Birmingham, Birmingham, UK; 2 Department of Cell Biology, SUNY Downstate Medical Center, Brooklyn, NY, USA

**Keywords:** Bleeding, genes, inherited thrombocytopenia, mutations, platelets, SLFN14

## The Schlafen (SLFN) Family

**Figure 1.**
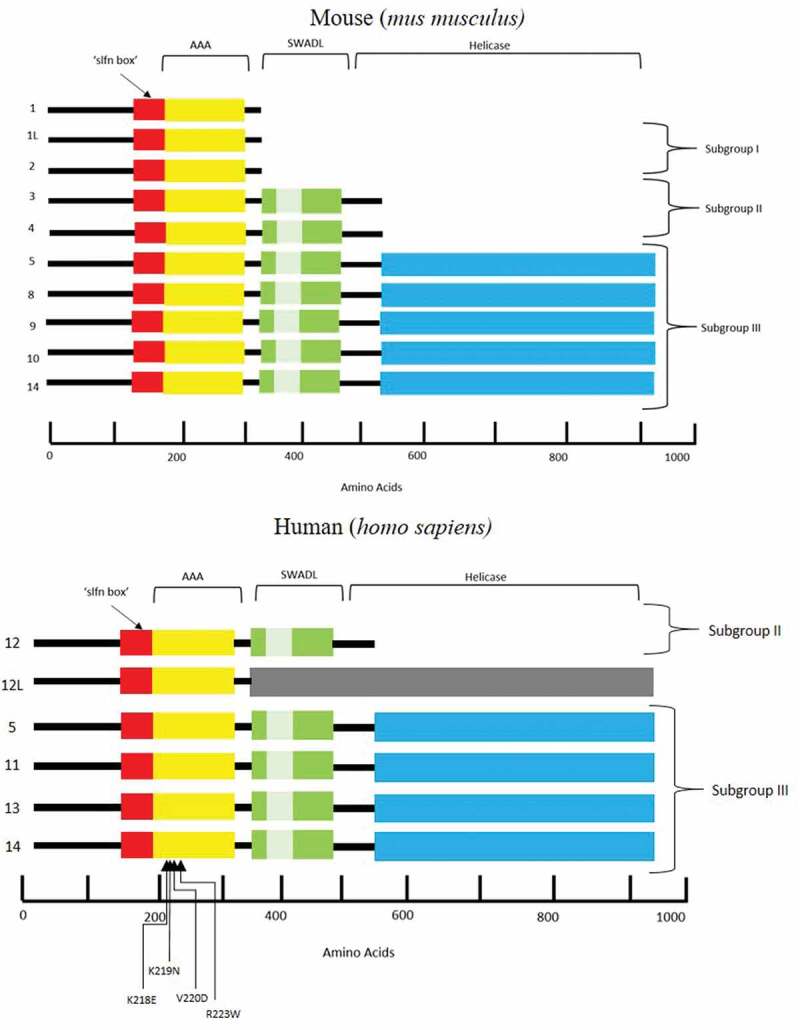
Schematic representation of the protein structure encoded by the *SLFN* family of genes, highlighting domains and regions in both *mus musculus* and *homo sapiens.* *‘*slfn’ box is unique to SLFN proteins and function remains unknown. The AAA domain is responsible for DNA and RNA metabolism and the SWADL region, again believed to be SLFN specific, is a sequence flanked by SWA and DL amino acids. The helicase regions at the C-terminal end of the protein are known to mediate DNA and RNA metabolism. The involvement of mutations and thrombocytopenia within the AAA domain of the SLFN14 protein remains unclear.

The *Schlafen* (*SLFN*) family of genes, which is limited to mammals, was initially discovered in mice by Schwarz et al., investigating the T cell lineage, regulating differentiation and in some instances ablating growth [1]. In particular, overexpression of *SLFN1* resulted in a cell cycle arrest at the G0/G1 stage. Therefore, the family had been named Schlafen, which is translated from German as “to sleep” [1]. Subsequent studies have classified the *SLFN* family into three distinct subgroups based on gene/protein size and domain homology [2,3]. Ten mouse and six human *SLFN* genes have been identified to date (Figure 1) [4]. All 10 mouse SLFN proteins possess a core region containing a unique “slfn box” motif with an unknown function. Subgroups II and III contain an extra domain at the C-terminus of the slfn box, conserved by the flanking five amino acid signature (Ser-Trp-Ala … -Asp-Leu) ‘SWA … DL’ which appears to be SLFN specific. This was discovered in early characterization of the SLFN family with SLFN3 and SLFN4 (members 3 and 4) possessing a 200 amino acid sequence not found in those in subgroup I [1]. Adjacent to the slfn box is the ‘**A**TPases **a**ssociated with diverse cellular **a**ctivities’ (AAA) domain. Based on protein homology studies, the AAA motif is thought to function similarly to classical AAA domains with the role of ATP/GTP binding in the course of DNA and RNA metabolism and therefore playing a fundamental role in the production and function of SLFN proteins [5,6]. Another protein was discovered with significant similarity to those in subgroup II, extending a further 400 amino acids towards the C-terminus terminal tail. The sequence was aligned by BLAST and the first 570 amino acids were homologous to SLFNs 3 and 4 while the remainder was unique to named SLFN8 leading to the classification of the final SLFN subgroup, III [2]. Additional homologous genes were identified as *SLFN5, SLFN9, SLFN10* and *SLFN14*, whereby the last two coding exons correspond to this unique subgroup. NCBI conserved domain database (CDD) searches revealed significant homology of subgroup III specific extension to motifs typical in superfamily I of RNA/DNA helicases which are known to mediate DNA and RNA metabolism [2]. The same characterization method applies to the human-specific *SLFN* genes with the absence of any subgroup I members and structural difference in S*LFN12L (SLFN12-LIKE)*. Notably, *SLFN5* and *SLFN14* are the only *SLFN* family genes present in both human and mouse. The predicted transcript of *SLFN12L* is of a similar length to those in subgroup III; however, it also encompasses sequences which are unaccounted for and based on this structural analysis, has not yet been assigned a subgroup. According to length and homology, SLFN14 belongs to subgroup III (Figure 1) [7]. The recently discovered crystal structure of rat SLFN13 (related to human SLFN13 and mouse SLFN8) N-domain gives a functional insight into its ability to cleave RNA acting as an endoribonuclease in inhibiting protein synthesis [8]. Importantly, SLFN13 is the closest paralogue for SLFN14 (44% identity and 61% similarity).

*SLFN14* in mice is located on chromosome 11 and comprised of five coding exons and in humans is on chromosome 17 with four coding exons [9]. *SLFN* genes are highly conserved throughout the mammalian species and are located in regions with other genes attributable to T-cell and macrophage development [1]. Early mouse knock-out studies investigating the role of *SLFN1* in the immune response showed no phenotype, suggesting potential functional redundancy or minimal contribution of some members in the process [9]. The precise mechanisms of how some of the specific members of the *SLFN* family cause human disease is still unclear.

## SLFN14 Mutations in Patients with Platelet Disorders and Bleeding

Platelet function disorders are associated with excessive bleeding and with the advent of next-generation sequencing (NGS) including whole exome sequencing (WES) the genetic impact of affected individuals can be evaluated. Initially, three extended families were investigated in the Genotyping and Phenotyping of Platelets Study, with three heterozygous single nucleotide variations in *SLFN14* resulting in missense mutations of consecutive amino acids in the AAA domain [10]. The following year, an additional family was discovered with another missense mutation in the same region (R223W) and in 2019, Saes et al., reported a further patient with an alternative base change at nucleotide c.657 yet still resulting in the K219N mutation (Table I and Figure 1) [11,12]. All four mutations were inherited in an autosomal dominant fashion whereby amino acid changes are conserved across all mammalian species, except for V220D. The R223W variant is the only variant out of these families to be reported on gnomAD (genome aggregation database) with a frequency of 6.5e-6. This highlights variant rarity amongst different populations and confinement to the families described. All individuals had macrothrombocytopenia, characterized by a reduced platelet count (below 150x10^9^/L) and enlarged platelets suggesting this is a typical feature of *SLFN14* related thrombocytopenia. Patients from the three initial families showed a platelet function defect with impaired aggregation to ADP, protease-activated receptor-1 (PAR-1) and Collagen and reduced ATP secretion from dense granules after PAR-1 stimulation [10]. Platelet aggregation was not stated for the cases in family D or E but an unspecified secretion defect in the latter case was reported [12]. The proband in family A was 31 years of age at the time of measurement and had experienced severe cutaneous bruising, prolonged bleeding from wounds and menorrhagia [10]. These symptoms were consistent across the three generations and are all typical of moderate thrombocytopenia. The proband in family B was 35 years old and had a history of spontaneous epistaxis; her mother was also recruited but had not experienced any prior bleeding tendencies [10]. Family C index case was 3 years old at the time, displayed a very low platelet count and reported easy bruising since birth [10]. Patients with variant R223W also reported bleeding symptoms after dental surgery and mild menorrhagia, typical of thrombocytopenia [11]. Individual history for the patient in family E was not detailed in publication however the patient was recruited to the study based on bleeding tendency so, therefore, is likely to be similar to those with the same mutation [12].

**Table I.** SLFN14 variants reported to date in patients with inherited macrothrombocytopenia, platelet-type bleeding disorder 20 (BDPLT20).

Patient	Genomic Variant	Protein effect	Variant type	Inheritance	Platelet count (x10^9^/L)	Mean platelet volume (MPV fl)	ISTH BAT Score	Aggregation/Secretion Defect	Reference
A; III 2	c.659 T > A	p.V220D	Missense	Het	140	9.1	5	ADP, Collagen and PAR-1-activating peptide/ATP	[10]
A; III 3	c.659 T > A	p.V220D	Missense	Het	74	10.4	10	ADP, Collagen and PAR-1-activating peptide/ATP	[10]
A; IV 2	c.659 T > A	p.V220D	Missense	Het	110	9.3	13	ADP,Collagen and PAR-1-activating peptide/ATP	[10]
A; IV 4*	c.659 T > A	p.V220D	Missense	Het	100	11.1	22	ADP, Collagen and PAR-1-activating peptide/ATP	[10]
A; IV 5	c.659 T > A	p.V220D	Missense	Het	116	11.2	21	ADP, Collagen and PAR-1-activating peptide/ATP	[10]
B; I 2	c.657 A > T	p.K219N	Missense	Het	83	11.9	13	ADP, Collagen and PAR-1-activating peptide/ATP	[10]
B; II 3*	c.657 A > T	p.K219N	Missense	Het	68	11.9	20	ADP, Collagen and PAR-1-activating peptide/ATP	[10]
C; II 2*	c.652 A > G	p. K218E	Missense	Het	89	13.0	NA	ADP, Collagen and PAR-1-activating peptide/ATP	[10]
D; II 1	c.667 C > T	p. R223W	Missense	Het	87	12.1	5	NA/NA	[11]
D; II 2	c.667 C > T	p. R223W	Missense	Het	91	21.0	2	NA/NA	[11]
D; III 3*	c.667 C > T	p. R223W	Missense	Het	79	12.3	9	NA/NA	[11]
E; I 1*	c.657 A > C	p.K219N	Missense	Het	NR	NR	NR	NA/NR	[12]

All patients identified with variants in the *SLFN14* gene and affected by inherited bleeding. *:Proband in family case; Het: Heterozygous inheritance pattern; International Society on Thrombosis and Haemostasis Bleeding Assessment Tool (BAT) score; NA: Not Available for *in vitro* study; NR: Not Reported in publication. Thrombocytopenia was defined as platelet count <150x10^9^/L

All patients in these reported families were heterozygous for the mutations and therefore phenotypically defined by platelet-type bleeding disorder 20 (BDPLT20) which is specific to heterozygous mutations in the *SLFN14* gene. The absence of homozygous patients in the families suggests the mutant allele in each variant group has significant influence over the wild type. Indeed, heterozygous SLFN14 protein expression was reduced by 65%-80% when compared to controls and in platelet spreading assays there is a reduction of proplatelet extensions from developing megakaryocytes [10,11]. This apparent dominant negative effect on overall protein expression can impact the maturation of megakaryocytes, affecting differentiation and production of fully functional platelets [10,11]. In order to decipher the role of *SLFN14* in platelet biogenesis, it has been demonstrated that SLFN14 acts as an endoribonuclease in controlling gene expression [13,14]. SLFN14 is strongly overexpressed in rabbit reticulocyte lysate where it is one of the major ribosome-associated proteins [13]. SLFN14 also binds to ribosomes in different model cell lines [14]. Upon binding, SLFN14 cleaves ribosomal RNA and ribosome-associated messenger RNA resulting in ribosomal degradation and disrupting translation [13,14]. Endoribonucleases mediate effective mechanisms in regulating gene expression; however, mutant variants of ribosome-related proteins, such as SLFN14, may cause errors in ribosome homeostasis and subsequent hematopoietic lineage dysfunction [15,16]. For this reason, we can hypothesize that *SLFN14* contributes to improper platelet formation from the disrupted translation of their precursors, megakaryocytes, which then results in an inherited thrombocytopenia [14]. Given the structural similarity of *SLFN13* to other subgroup III genes (such as *SLFN14*) and the recent report on SLFN13’s role as an endoribonuclease it seems plausible to hypothesise that SLFN14 may be impacting megakaryocyte differentiation and platelet synthesis by also acting as an endoribonuclease, inhibiting translation and preventing complete protein synthesis within the hematopoietic lineage [8].

It is only with the help of new genetic approaches over recent years, that inherited thrombocytopenias with previously unknown causes can now be attributed to single genetic defects. This not only allows for expanding knowledge within the field of genetics and hematological disorders but also provides patients with a clear diagnosis and a plan for tailored clinical management.

## Main Findings

- Mutations in *SLFN14* cause inherited macrothrombocytopenia characterized by enlarged platelets and moderate to severe bleeding phenotypes.
- Three out of the five families with *SLFN14* genetic variants show reduced aggregation to agonists ADP, PAR-1 and Collagen, decreased ATP secretion and dominant inheritance pattern.
- Endoribonucleolytic activity of SLFN14 contributes to platelet formation by regulating translation process during their maturation.
- The exact role of *SLFN14* in platelet biogenesis and how mutations within its AAA domain contribute to inherited thrombocytopenia remain to be explored.
